# A retrospective study of 370 patients with oral lichen planus in Turkey

**DOI:** 10.4317/medoral.18356

**Published:** 2013-03-25

**Authors:** Birsay Gümrü

**Affiliations:** 1 Department of Oral Diagnosis and Radiology, Faculty of Dentistry, Marmara University, Istanbul, Turkey

## Abstract

Objectives: Although several detailed studies concerning the patient profile and clinical features of oral lichen planus have been undertaken all over the world in different populations, a similar study has not yet been conducted in a Turkish population. The purpose of this retrospective study was to describe the demographic and clinical characteristics of a group of patients with oral lichen planus in Turkey. Study Design: Charts of 370 patients, from the archive of Oral Diagnosis and Radiology Department of Marmara University Faculty of Dentistry (Istanbul, Turkey), with histologically confirmed clinical diagnosis of oral lichen planus in the period 1990-2010 were retrospectively reviewed. Results: Of the 370 patients, 260 (70.3%) were women and 110 (29.7%) were men. The mean age was 49.84±13.41 years (range of 16-83). The lesions were asymptomatic in 63 patients (17%). Nearly half of the patients (47.6%) had multiple sites of involvement. Predominantly red forms were the most frequent, affecting 60.5% of patients. Approximately 17% of the patients had symptoms of possible extraoral involvement. No evidence suggesting a connection between oral lichen planus and tobacco or alcohol use was found. Only one out of the 370 cases had histologically proven malignant transformation. Conclusions: The patient profile and clinical features of oral lichen planus in Turkey were generally similar to those described in other populations. The preponderance of the red forms and also the fact that majority of patients referred themselves to our clinic highlighted the lack of awareness among Turkish health care providers about lichen planus.

** Key words:**Oral lichen planus, clinical features, patient profile.

## Introduction

Oral lichen planus (OLP) is a relatively common chronic inflammatory disorder of middle aged and elderly, which seems to represent a spectrum of conditions that share a common background with clinical presentations ranging from mild painless white papular lesions to painful erosions and ulceration (1,2). The exact cause is unknown, but there is overwhelming evidence that cell-mediated immunity, possibly initiated by endogenous factors in those genetically predisposed to the development of the disease, is crucial in the pathogenesis.

Several detailed epidemiological and clinical investigations of OLP have been undertaken all over the world in different countries, such as Hungary (3), the United States (4-8), Denmark (9,10), Australia (11), Brazil (12,13), Spain (14-17), Israel (18), Italy (19), Sweden (20), Iran (21), United Kingdom (22), China (23). A general similarity in the nature of this disease has been confirmed in different populations - including a predilection for females, a mean age of onset in the fourth to fifth decades of life, and the buccal mucosa being the most common site. To the best of our knowledge, so far a similar study has not been conducted in a Turkish population.

The aim of this retrospective study was to evaluate general features and clinical presentation of OLP in a group of Turkish patients treated and followed in our clinic during the past 20 years and to describe similarities and differences of these patients relative to those in previously reported series in other populations.

## Material and Methods

The patient archive of Oral Diagnosis and Radiology Department of Marmara University Faculty of Dentistry (Istanbul, Turkey) was retrospectively reviewed for the period between 1990 and 2010 for charts of patients with histologically confirmed clinical diagnosis of OLP according to the diagnostic criteria of World Health Organization (WHO) of 1978 modified by van der Meij and van der Waal (24). Relevant retrospective data was selected and extracted systematically by a single observer (BG). The charts of patients with a diagnosis of lichenoiddysplasias or lichenoid lesions caused by an identifiable cause such as a hypersensitivity reaction to dental restorative materials (such as amalgam) or drugs (such as non-steroidal anti-inflammatory drugs and angiotensin-converting enzyme inhibitors), and charts that did not include histological confirmation of OLP were excluded from this study (number unknown). The design of this retrospective study was approved by the Clinical Research Ethics Committee of the Istanbul University School of Medicine, and patient anonymity was strictly respected.

A total of 370 charts were reviewed, information regarding age, gender, family history of lichen planus (first-degree relatives), sites of oral involvement, number of sites affected, chief symptoms, predominant clinical forms, extraoral involvement, presence of any systemic disease and use of any drugs, habits regarding tobacco and/or alcohol consumption, treatment provided (topical corticosteroid in mucosal adhesive paste or as intralesional injection, or systemic corticosteroid), side-effects of treatment, histologically proven malignant transformation at a previously diagnosed OLP site was obtained. Exacerbating factors of OLP identified by either patients or the examiner were also noted.

According to the description done at the time of diagnosis, the clinical forms of OLP were detailed and gathered in two categories: (i) predominantly white forms including papular, reticular or plaque presentations; and (ii) predominantly red forms including atrophic (erythematous) and erosive presentations with concomitant white lesions based on classification of Bagán Sebastián et al (14). In patients with more than one clinical type of lesion, such as reticular and erosive, the most severe form of the disease (i.e. erosive) was used to classify the lesions.

The majority of charts contained the required data for analysis. If necessary, patients were in due course re-contacted by telephone to revise and complete the information.

Statistical analysis was performed using NCSS 2007 (Number Cruncher Statistical System) and PASS 2008 (Power Analysis and Sample Size) (NCSS LLC Inc., Utah, USA). Statistical analysis was carried out with the chi-squared test and Student’s t-test for significance. A p value <0.05 was considered statistically significant.

## Results

A total of 370 charts of patients with confirmed diagnosis of OLP were retrospectively analysed, of whom 260 (70.3%) were women and 110 (29.7%) were men giving a female to male ratio of 2.36:1.

The mean age of the patients at presentation was 49.84±13.41 years, with an overall range of 16-83 years. OLP was most prevalent among women between ages of 50-59 years (34.2%). The highest prevalence for men was found in the age group 40-49 (30%).

The medical histories and medications reported by the patients are shown in  Table 1. Most of the patients were non-smokers (81.9%) and non-drinkers (98.7%).

**Table 1:**
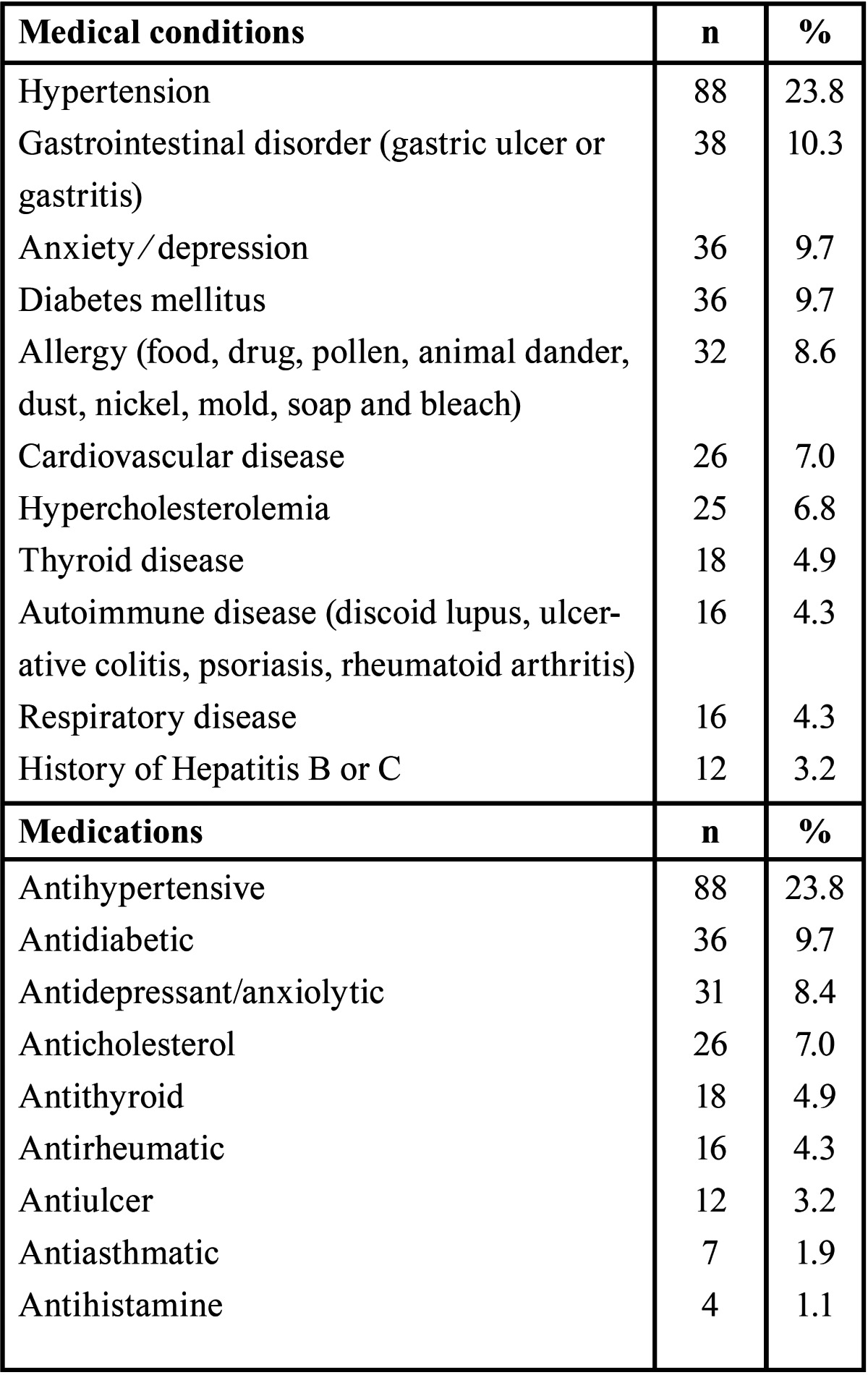
Table 1 . Medical conditions and medications reported by patients with oral lichen planus.

About 77.3% of the patients referred themselves to our clinic. Only 10.3% of the patients had been referred by their general dental practitioners, 5.7% by other dental specialities, 5.1% by dermatologists, 0.8% by their general medical practitioners and another 0.8% by ear-nose-throat specialists. Family histories of lichen planus were extremely rare (0.8%).

A total of 307 patients (83%) reported symptoms, whereas 63 (17%) were asymptomatic. Chief complaints of 370 patients with OLP at time of initial clinical presentation are shown in  Table 2.

**Table 2:**
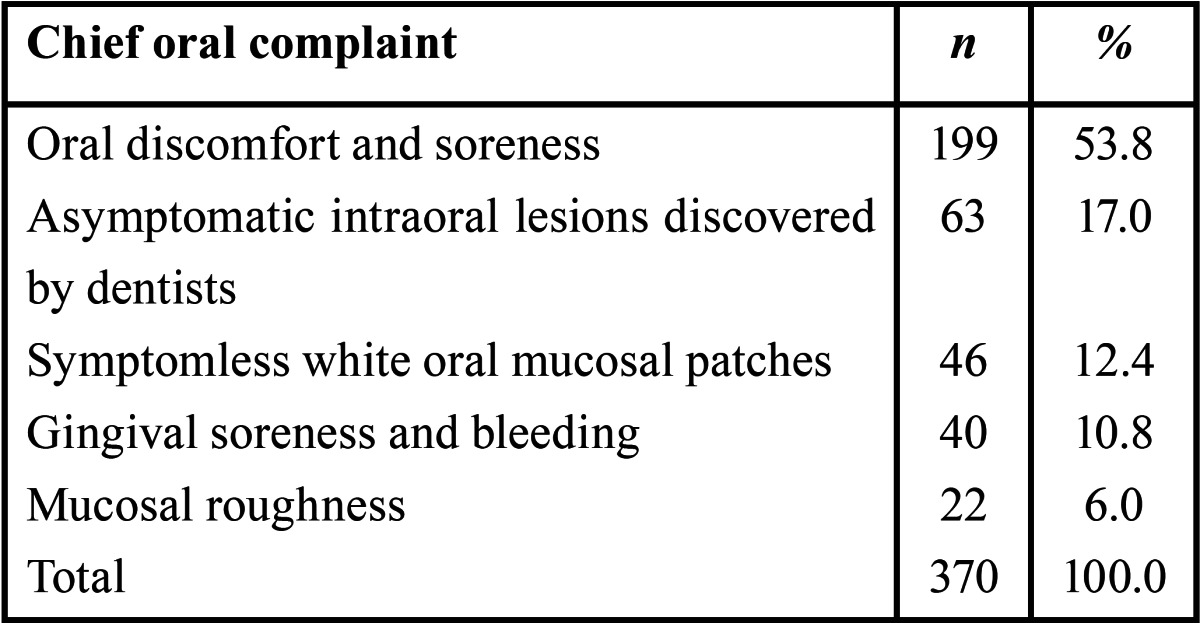
Chief complaints of 370 patients with oral lichen planus at time of initial clinical presentation.

Nearly half of the patients (47.6%) exhibited multiple sites of involvement, with the buccal mucosa being the most common site (88.1%), followed by tongue (27.6%), gingiva (25.9%), labial mucosa (8.1%), hard palate (7.8%), alveolar ridge (5.4%) and floor of the mouth (3%). Lesions on the soft palate (1.4%) and oropharynx (0.3%) were uncommon.

Regarding the clinical signs at initial presentation, the predominantly white forms were observed in 39.5% of the cases (146 patients), and red forms in 60.5% (224 patients) in this series.  Table 3 shows the relationship between the clinical forms and different variables.

**Table 3:**
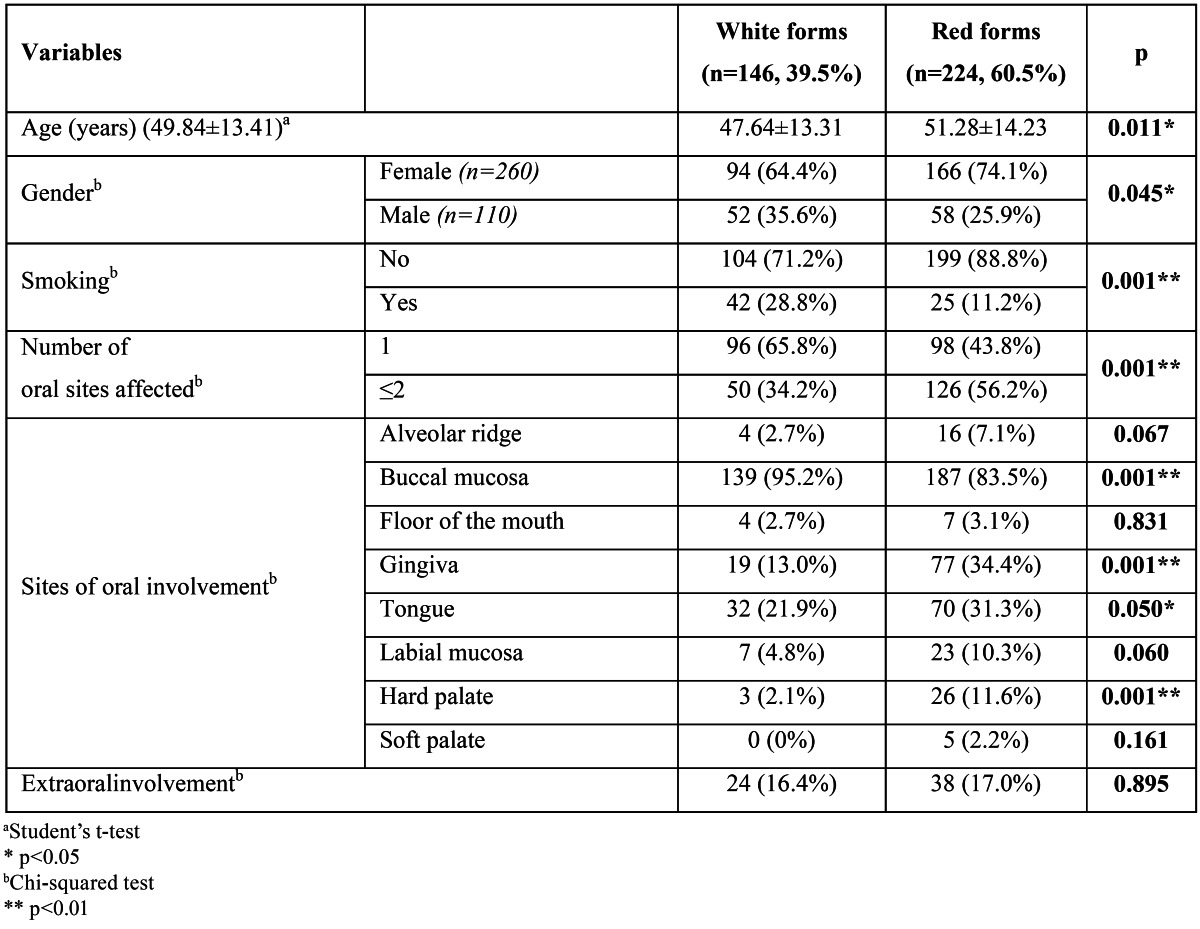
Relationship between the clinical forms of oral lichen planus and different variables.

Precipitating factors that resulted in an exacerbation of the disease including foods (most frequently tomatoes, citrus, and spicy items), stress, dental hygiene procedures and dentures were identified by patients. At least one of these factors was reported by nearly 53% of patients with foods identified most frequently (26.5%) followed by stress (20.3%).

Sixty two patients (16.8%) in the entire series had symptoms of possible non-oral lichen planus, or a history of specialist-diagnosed lichen planus affecting the skin or non-oral mucosal membranes; of those, 54 (14.6%) had skin involvement, 11 (3%) had genital involvement and 7 (1.9%) in other sites (e.g. scalp, nail). The age and gender distribution of the OLP patients with these lesions did not differ from those of without extraoral involvement.

Topical steroids alone were prescribed to 180 (48.6%) and in combination with systemic steroids to 4 (1.1%) of the 370 patients at the initial examination. The choice of drug depended on the severity of symptoms and patient preference (corticosteroid in mucosal adhesive paste or as intralesional injection). A hundred and thirty eight of the remaining patients (37.3%) were not treated (minimal or no symptoms), but were followed up with periodic oral examinations. In 48 (13%) patients, dermatological consultation was needed due to extraoral involvement. Treatment was undertaken usually with the goal of achieving complete control of symptoms with minimal side-effects. Patients receiving long-term maintenance therapy with topical steroids reported no systemic side-effects. However, oral candidiasis was an occasional complication, with 4 (2.2%) of the 184 patients, needing antifungal therapy at some point during the follow-up period.

Only in one out of the 370 OLP cases, histologically proven malignant transformation was documented on the buccal mucosa of an elderly woman at a site diagnosed as erosive lichen planus two years previously. This patient had a history of neither tobacco nor alcohol use.

## Discussion

Clinical manifestations of OLP have been studied in various populations. The interest in studying this disease is due to its relative frequency, the presence of symptoms, the lack of an effective cure of the lesions and in addition the concern of a risk of malignant transformation (12).

Retrospective surveys, such as ours, have many limitations and cannot be compared satisfactorily to prospective studies. However, they are useful in evaluating patient populations. When interpreting the results, we need to keep in mind that our clinic is a tertiary referral clinic, and the study sample reflects the findings in this selected group of patients.

Because there are no universally accepted specific diagnostic criteria for OLP, in a majority of studies the diagnosis was solely based on clinical findings, and histopathological examination was performed in case of suspicion (3,9,11-13,22) not taking into account other conditions presenting a similar clinical appearance, such as leukoplakia, lupus erythematosus and even squamous cell carcinoma. In addition, histopathological assessment of OLP was demonstrated to be rather subjective in a study (25). Thus, some oral lesions diagnosed clinically or histologically as OLP in previous reports might actually have been lichenoiddysplasias, premalignant dysplasias with lichenoid appearances. Therefore, the latest criteria proposed by van der Meij and van der Waal (24) based on the 1978 clinical and histopathological definition of OLP by the WHO was used in the present study similar to a few previous studies (17,19,23).

A number of different clinical classifications of OLP have been proposed (4,9). In the present study, practical considerations led us to classify OLP in two groups being predominantly white forms including papular, reticular or plaque presentations and predominantly red forms including atrophic (erythematous) and erosive presentations with concomitant white lesions as suggested by Bagán Sebastián et al (14). Previously, this classification was used by other authors (16,19).

The profile of our OLP patients was generally similar to that found in other studies (4-11,13,14,16-23). The disease was more prevalent among women more than twice as men, developed at an earlier age in men, lacked a familial pattern, and the patients’ mean age was approximately 50 years. As previously reported by others, we also found no evidence suggesting a connection between OLP and tobacco or alcohol use (3-5,10,13,15,18,19). However, plaque type lesions were found significantly more frequently among smokers than among non-smokers, consistent with the study of Thorn et al (10).

Clinically the most common location of OLP is the buccal mucosa, followed according to some authors by the tongue (8,9,12-15,17,19,21,22) or, according to others by the gingiva (4-6,16,18). In the present study the buccal mucosa was found to be the most common site (88.1%), followed by tongue (27.6%) and gingiva (25.9%).

Compared to most of the previous investigations, a preponderance of the red forms of OLP was found in the present study similar to few previous studies (5,6,9,14,20). This can probably best be explained by referral of patients with red forms of OLP to tertiary clinics like ours related to difficulties in diagnosis and symptoms of pain.

When the two OLP groups were compared, statistically significant differences were observed in some instances. Patients with red forms of OLP showed a statistically higher mean age than those with white forms. In addition, white lesions presented a much greater male predominance while red lesions presented a much greater female predominance. These tendencies have been pointed out in a few studies (7,14). Location of the lesions within the oral cavity also showed intergroup differences. The buccal mucosa was the only region where white forms clearly predominated over red forms; the latter prevailed in other oral locations such as gingiva, tongue and hard palate. Similar findings were pointed out by Bagán Sebastián et al (14). Our results showed that patients with red forms had a higher number of sites affected, which is similar to the results reported by others (14,15,23).

Patients with OLP may have concomitant disease in one or more extraoral sites. Extraoral involvement can precede, arise concurrently with or appear after the development of OLP (22). In the present study, 16.8% of patients had a history of symptoms of possible non-oral lichen planus. However, different studies reported a higher incidence (up to 45%) (5,9,10,13). This discrepancy perhaps may be explained by patients being evaluated to uncover potential sites of extraoral involvement at the time of diagnosis of OLP without considering the past medical history. The concomitant involvement, even occasional, in other body sites highlight the importance of having patients with OLP evaluated by a multidisciplinary group of health care providers. Unfortunately, the finding that 77.3% of the patients in our study population referred themselves to our clinic shows the lack of awareness about OLP among Turkish dentists and physicians who are not familiar with the importance of identifying and referring patients with oral lesions of lichen planus for professional follow-up because of the possibility of a malignant potential of this condition.

Because there is no definite mode of treatment for a complete and lasting cure of OLP, the main consideration is the satisfactory control of the disease. In general, treatment is only directed towards symptomatic cases those usually exhibit the red forms of the disease. For asymptomatic lesions, no medication is required but the patient should be informed of the presence and nature of the condition and reviewed clinically on a regular basis. Administration of topical and/or systemic corticosteroids has been the most widely used treatment for OLP and proved to be the therapy of choice for the symptomatic cases in this study. Topical corticosteroids can be effective for initial treatment for maintaining a level of control compatible with a good quality of life for many patients. When systemic corticosteroids are indicated we prefer to treat the patients in cooperation with their physicians because potential adverse side effects are anticipated. Because candidiasis can complicate OLP during corticosteroid treatment, the use of appropriate antifungal agents is important for optimal control. In our study, candidiasis was identified in 4 patients, all of whom were women with red forms of OLP affecting multiple oral sites.

Oral lichen planus tends to follow an evolution that comprises periods of remission and exacerbation on a chronic course that might lead to malignant transformation. Nevertheless, the potential for malignant transformation of OLP remains controversial. Reportedly, 0.4% up to 2.5% of patients with OLP has oral malignancy at the site of the OLP lesion with a special high risk in the erosive and atrophic forms (3-5). Malignant transformation was observed in only one out of 370 OLP cases during the observation period in the present study. This developed on the buccal mucosa, which is regarded as a low risk zone for the development of cancer, of an elderly woman with pre-existing erosive OLP lesions with no history of any known risk factors (alcohol or tobacco habit).

In conclusion, the results of this retrospective survey revealed that the profile and clinical features of OLP patients in Turkey were generally similar to those described in other populations. Compared to most of the previous investigations, the preponderance of the red forms of OLP in the present study and also the fact that majority of patients referred themselves to our clinic highlighted the remarkable lack of awareness among Turkish dentists and physicians about OLP, requiring a multidisciplinary approach of health care providers.
